# Chronic high‐fat diet induces multi‐organ dysfunction and metabolic homeostasis disruption in *Macaca fascicularis*

**DOI:** 10.1002/ame2.70124

**Published:** 2026-01-14

**Authors:** Hongyi Chen, Wei Liu, Dan Zhou, Shuhua Liu, Yalun Guan, Zongyu Miao, Lei Cai, Xuejiao Li, Yunfeng Li, Zhongqiang Huang, Yi Jin, Ge Li, Yu Zhang

**Affiliations:** ^1^ Guangdong Provincial Biotechnology Research Institute (Guangdong Provincial Laboratory Animals Monitoring Center) Guangzhou China; ^2^ Guangzhou Huazhen Biosciences Co., Ltd. Guangzhou China; ^3^ Department of Geriatrics Shenzhen People's Hospital (The Second Clinical Medical College, Jinan University, The First Affiliated Hospital, Southern University of Science and Technology; Shenzhen Clinical Research Center for Geriatrics; Guangdong Provincial Clinical Research Center for Geriatrics) Shenzhen China; ^4^ Shenzhen Institute for Drug Control (Shenzhen Testing Center of Medical Devices) Shenzhen China; ^5^ Guangzhou National Laboratory Guangzhou China

**Keywords:** animal model, *Macaca fascicularis*, metabolic dysfunction, proteome

## Abstract

**Background:**

The aim of the study was to develop a non‐human primate model of metabolic dysfunction in *Macaca fascicularis* using chronic high‐fat diet (HFD) to mimic clinical disease progression.

**Methods:**

Thirty‐five male macaques aged 10–15 years underwent an 18‐month HFD intervention. Physiological parameters (BMI, BP, hematology), liver fat fraction (evaluated by ultrasound/MRI), cardiac function (assessed by echocardiography), and histopathology (using liver biopsy) were measured before and after the intervention. Serum proteomics with KEGG/STRING analyses identified molecular mechanisms.

**Results:**

Within 6 months, HFD induced dyslipidemia (elevated TG, TCHO, HDL‐C, LDL‐C). After 18 months, metabolic dysfunction‐associated steatohepatitis (MASH) was confirmed by histopathology in 57.14% (16/28) of macaques, diabetes (elevated FPG/HbA1c) in 17.86% (5/28), and myocardial hypertrophy (elevated LVMass/LAD) in 46.43% (13/28). Proteomics identified Bile acid‐CoA: amino acid N‐acyltransferase (BAAT) as a MASH hallmark protein, the level of which was inversely correlated with the degree of fibrosis. For diabetes, citrate synthase (CS) and malate dehydrogenase 1 (MDH1) impaired glucose oxidation via the TCA cycle, while hexose‐6‐phosphate dehydrogenase (H6PD) disrupted gluconeogenesis. Myocardial hypertrophy was associated with the downregulation of SRC proto‐oncogene, non‐receptor tyrosine kinase (SRC), mitogen‐activated protein kinase 14 (MAPK14), emerin (EMD), and integrin subunit beta 1 (ITGB1).

**Conclusions:**

An 18‐month HFD successfully established a translational *M. fascicularis* model replicating key metabolic disorders (MASH, diabetes, cardiac hypertrophy). BAAT, CS/MDH1/H6PD, and SRC/MAPK14/EMD/ITGB1 were identified as mechanistic biomarkers for these conditions.

## Graphical abstract

An 18‐month HFD successfully established a translational *Macaca fascicularis* model replicating key metabolic disorders (MASH, diabetes, cardiac hypertrophy). MASH was determined by liver biopsy histology, the presence steatosis, inflammatory infiltration, hepatocytic ballooning, and fibrosis were considered as MASH; diabetes was diagnosed according to levels of fasting blood glucose and glycated hemoglobin (HbA1c), it defined by fasting blood glucose ≥7.0 mmol/L and glycated hemoglobin (HbA1c) ≥6.5%; and myocardial hypertrophy was identified based on increased (≥20%) Left Ventricular Mass (LVMass) measured by echocardiography. Base on serum proteomics, BAAT, CS/MDH1/H6PD, and SRC/MAPK14/EMD/ITGB1 were identified as mechanistic biomarkers for these metabolic disorders.

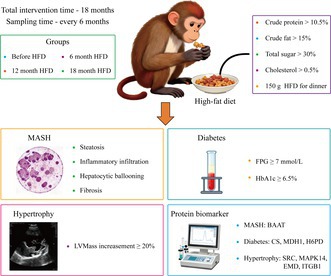

## 1 INTRODUCTION

Chronic high‐fat diet (HFD) intervention can lead to metabolic disorders, causing multiple organ dysfunction and metabolic homeostasis disorders, thereby contributing to the development of metabolic syndrome (MetS). MetS is a multifactorial metabolic disorder primarily manifested as central obesity, hyperglycemia, hypertension, and dyslipidemia.^1^ It serves as an important pathological basis and is considered an early stage of major non‐communicable diseases such as type 2 diabetes mellitus, cardiovascular disease, and cerebrovascular disease. Statistically, the prevalence of MetS is significant, with approximately one‐quarter of the adult population in the United States affected.^2^ Applying the same growth rate as in the US, the prevalence of MetS in China was estimated to be 15.5% in 2017.^3^ Diagnostic criteria for MetS vary and include those set by the WHO,^4^ the ATP III,^5^ the AHA/NHLBI,^2^ and the IDF.^6^ According to the IDF criteria, MetS is diagnosed when an individual has central obesity (measured by a high waist circumference) plus at least two of the following: high triglyceride (TG) levels; low high density lipoprotein cholesterol (HDL‐C) levels; hypertension; and high fasting plasma glucose (FPG) levels.^7^

MetS ranks among the leading causes of global mortality, yet its pathogenic mechanisms remain only partially elucidated. Developing animal models that closely mirror the features of human MetS is of paramount importance for fundamental research into its pathological mechanisms and the discovery of potential intervention drugs.^8^ At present, two primary strategies are employed for creating metabolic dysfunction animal models. Among these, single‐gene animal models (characterized by mutations in a single gene) and diet‐induced obesity models are the most prevalent.^9^ The diet‐induced obesity model closely mimics the etiological pathways of human obesity and MetS. By simulating high‐calorie diets and sedentary lifestyles, this model effectively recapitulates the pathological hallmarks of human MetS.^10^ Among them, the induction methods include HFD,^11^ high‐starch and high‐sugar induced diets ,^12^ and choline‐methionine deficiency.^13^ In contrast, single‐gene models have often been favored for shorter‐term experiments since they obviate the need for extended feeding regimens to induce obesity.^14^ As a result, fewer specimens are required, but high mortality rates also increased research costs.^15^ Owing to inherent physiological disparities between species, the translational potential of animal model research is often limited.

To align with clinical pathogenesis and disease progression, this study employed non‐human primates (NHPs) with optimized HFD protocols to mitigate appetite suppression. Metabolic dysfunction model validation utilized BMI, blood pressure (BP), hematological indices, and histopathological examinations against established clinical diagnostic criteria. Bioinformatics analysis of proteomics data verified whether the model accurately recapitulated clinical features to facilitate subsequent experiments and drug testing. A correlation analysis between cardiac function indices and metabolic parameters elucidated their complex interrelationships in NHPs.

## 2 MATERIALS AND METHODS

### 2.1 Animal preparation

Thirty‐five male *Macaca fascicularis* aged 11–15 years were included in this study. Comprehensive evaluations, including weight and BP measurement, and blood biochemical analysis, histopathology analysis were systematically conducted. All procedures adhered to the ‘Care and Use of Laboratory Animals’ guidelines and were approved by the Institutional Animal Care and Use Committee of Guangdong Provincial Biotechnology Research Institute (IACUC no. 2021147, AAALAC accredited).

### 2.2 Animal welfare monitoring and humane endpoints

During the 18‐month study, daily health checks, weekly assessments of body weight and food intake, and regular blood biochemical analyses were conducted. Furthermore, we also explicitly defined the predetermined humane endpoint criteria. These criteria, established in consultation with veterinary staff, are based on objective measures such as severe weight loss (>15%–20%), prolonged anorexia, signs of profound weakness or distress, and the development of untreatable conditions. They were rigorously followed.

### 2.3 Intervention methods for HFD

A HFD was produced using lard, yolk powder, cholesterol, sucrose and standard maintenance diet. This diet was obtained in the form of pellets from Guangzhou Guolong Technology Co., Ltd. The HFD must ensure basic nutrition, with the content of crude protein being more than 10.5%, crude fat more than 15%, total sugar more than 30%, and cholesterol more than 0.5%. *Macaca fascicularis* were feed three times a day, 100 g standard maintenance diet for breakfast, 150 g fruit for lunch, and 150 g HFD for dinner.

### 2.4 Weight measurement and body mass index (BMI) calculation

*Macaca fascicularis* were fasted for 12 h. After anesthesia, the animals' weights and their body lengths from the top of the head to the tail root (the crown‐rump lengths) were measured. The average of three measurements was taken and the BMI was calculated using the formula: BMI (kg/m^2^) = weight/crown‐rump length.^2^

### 2.5 BP measurement

Blood pressure was measured within 20 min of anesthesia induction in the animals, ensuring that the BP monitor was at the same level as the heart and brain. The average of two measurements was taken.

### 2.6 Serum biochemistry

*Macaca fascicularis* were fasted for 12 h, and procoagulant vacuum blood collection tubes were used for collected blood samples from the hind limb vein. The blood sample was centrifuged at 3000 rpm for 10 min to collect serum. An automated biochemical analyzer (Hitachi, 3100, Japan) was used to detect blood biochemical indicators.

### 2.7 Qualitative and quantitative analysis of liver fat

*Macaca fascicularis* were fasted for 12 h, ultrasound imaging was performed to visualize and assess the liver and renal parenchyma. The grayscale intensity of the liver and kidney parenchyma was measured using ImageJ.

After ultrasound imaging, the *M. fascicularis* was placed in a supine position on the scanning bed of the MRI equipment. It was adjusted to the appropriate position after anesthesia. Transverse MRI was performed using a 3D FACT imaging sequence. The liver was divided into eight segments according to the Couinaud classification. The regions of interest (ROI) with an area of 10–30 mm^2^ were defined. The fat fraction values for each liver segment were determined by averaging the fat fractions measured from the 3 ROIs in each segment.

### 2.8 Liver pathology staining

Polyformaldehyde‐fixed tissues were processed through graded ethanol, cleared with xylene and then infiltrated with molten paraffin. Liver tissues were sectioned on a rotary microtome (Leica, RM2235, Germany) at 5 μm thickness and mounted on glass slides. Slides were stained for hematoxylin and eosin (H&E) and Sirius Red, respectively. H&E staining was used for steatosis and lobular inflammation scores, and Sirius Red staining was used to score hepatic fibrosis.

### 2.9 Echocardiography examination

After animal anesthesia, two‐dimensional, pulsed‐wave Doppler, and tissue Doppler echocardiography scans were performed using an ultrasound diagnostic device (Vivid iq, GE Healthcare, USA). All parameters of cardiac structure and function were obtained from three consecutive cardiac cycles, and the measured parameters were then averaged.

### 2.10 Proteomics of serum

Serum was denatured, reduced, alkylated, desalted (C18 column), quantified (Bradford), and separated by SDS‐PAGE on 12% gel. A 20 μg sample was separated on a Gemini C18 column (5 μm, 20 cm × 180 μm) using a Shimadzu LC‐20 AD system with high‐pH mobile phases. The eluted peptides were collected into 10 fractions, pooled, dried, and reconstituted. The peptides were separated on C18 trap column (1.8 μm, 25 cm × 75 μm) using a Bruker nanoElute system with low‐pH ACN gradient (300 nL/min). Peptides were ionized via a CaptiveSpray source and analyzed using a timsTOF Pro2 mass spectrometer in DIA mode.

DIA data were processed using Spectronaut (v17) with mProphet FDR estimation (FDR ≤ 1%). Differentially Expressed Proteins (DEPs) were identified using MSstats (quantile normalization; criteria: fold‐change ≥2, *p*‐value < 0.05). Protein annotation and pathway analysis were performed using KEGG and STRING databases.

### 2.11 Statistical analysis

Statistical analyses were conducted using GraphPad Prism (version 8, GraphPad Inc., San Diego, CA, USA) and SPSS 27.0. Data were represented as the mean ± standard deviation (SD). A Wilcoxon signed rank test or Mann–Whitney test was employed to compare the changes at baseline (before HFD), and after 18 months of HFD. The correlation between changes in echocardiographic indices and metabolic parameters was assessed using Pearson correlation analysis. Multiple regression analysis was performed to predict various echocardiographic indices and metabolic parameters. A *p*‐value less than 0.05 was considered statistically significant.

## 3 RESULTS

### 3.1 Prolonged HFD intervention can result in obesity and metabolic disorders

**FIGURE 1:**
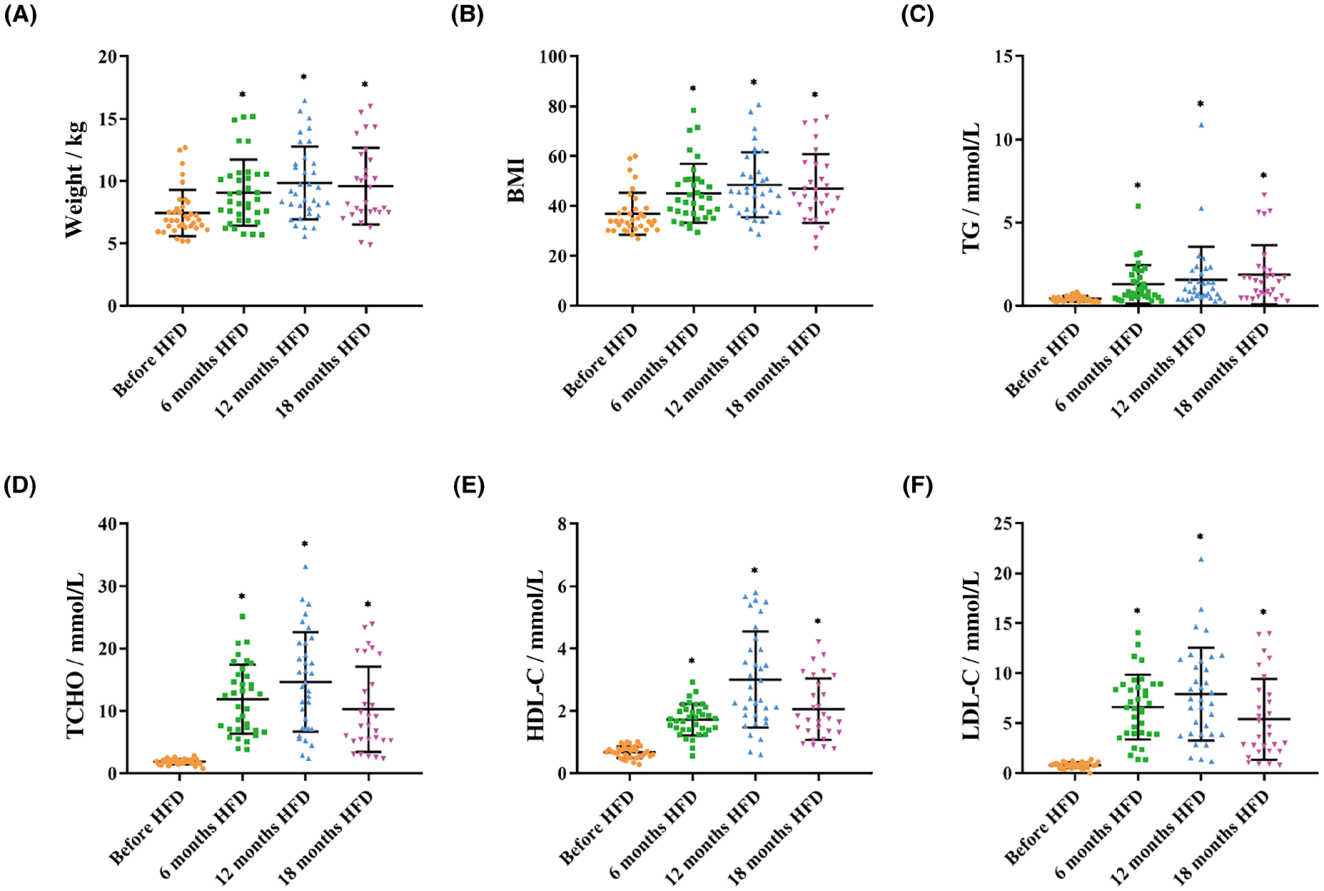
Body weight and blood lipids changes induced by HFD. After the HFD intervention, weight (A), BMI (B), TG (C), TCHO (D), HDL‐C (E) and LDL‐C (F) were significant increased in serum biochemistry analysis. Data are presented as mean ± SD. Statistical significance: *p < 0.05.

Physiological indicators in *M. fascicularis* were longitudinally monitored at 6‐month intervals throughout the 18‐month HFD intervention period. Quantitative analysis revealed statistically significant increases in weight and BMI after the intervention, indicating overweight and obesity. Furthermore, the HFD induced changes in blood lipid profiles. After 6 months of intervention, significant increases were observed in TG, total cholesterol (TCHO), HDL‐C, and low density lipoprotein cholesterol (LDL‐C) levels. At the three time points of 6 months (*n* = 35), 12 months (*n* = 34), and 18 months (*n* = 28) after HFD intervention, the increases in body weight, BMI, TG, TCHO, HDL‐C, and LDL‐C were as follows: body weight increased by 22.46%, 31.82%, and 28.34%; BMI by 21.63%, 33.23%, and 27.98%; TG by 197.67%, 262.79%, and 332.56%; TCHO by 539.25%, 687.10%, and 451.08%; HDL‐C by 155.22%, 349.25%, and 205.97%; and LDL‐C by 741.77%, 901.26%, and 582.28%, respectively (Figure 1A–F, Tables S1 and S2). These indicators fluctuated over time, but the overall trend of change remained consistent, with a relatively small amplitude of fluctuation, indicating that the changes in these indicators have reached a plateau. Metabolic syndrome criteria require concurrent obesity (BMI ≥ 40 kg/m^2^), hypertension (systolic BP ≥ 140 mmHg or diastolic BP ≥ 90 mmHg), and dyslipidemia (TG ≥ 1.7 mmol/L, TCHO ≥ 6.2 mmol/L, or LDL‐C ≥ 4.1 mmol/L). These indicators thus suggest metabolic syndrome in *M. fascicularis* after 18 months of HFD intervention.

### 3.2 Prolonged HFD intervention induces the progression to MASH

**FIGURE 2:**
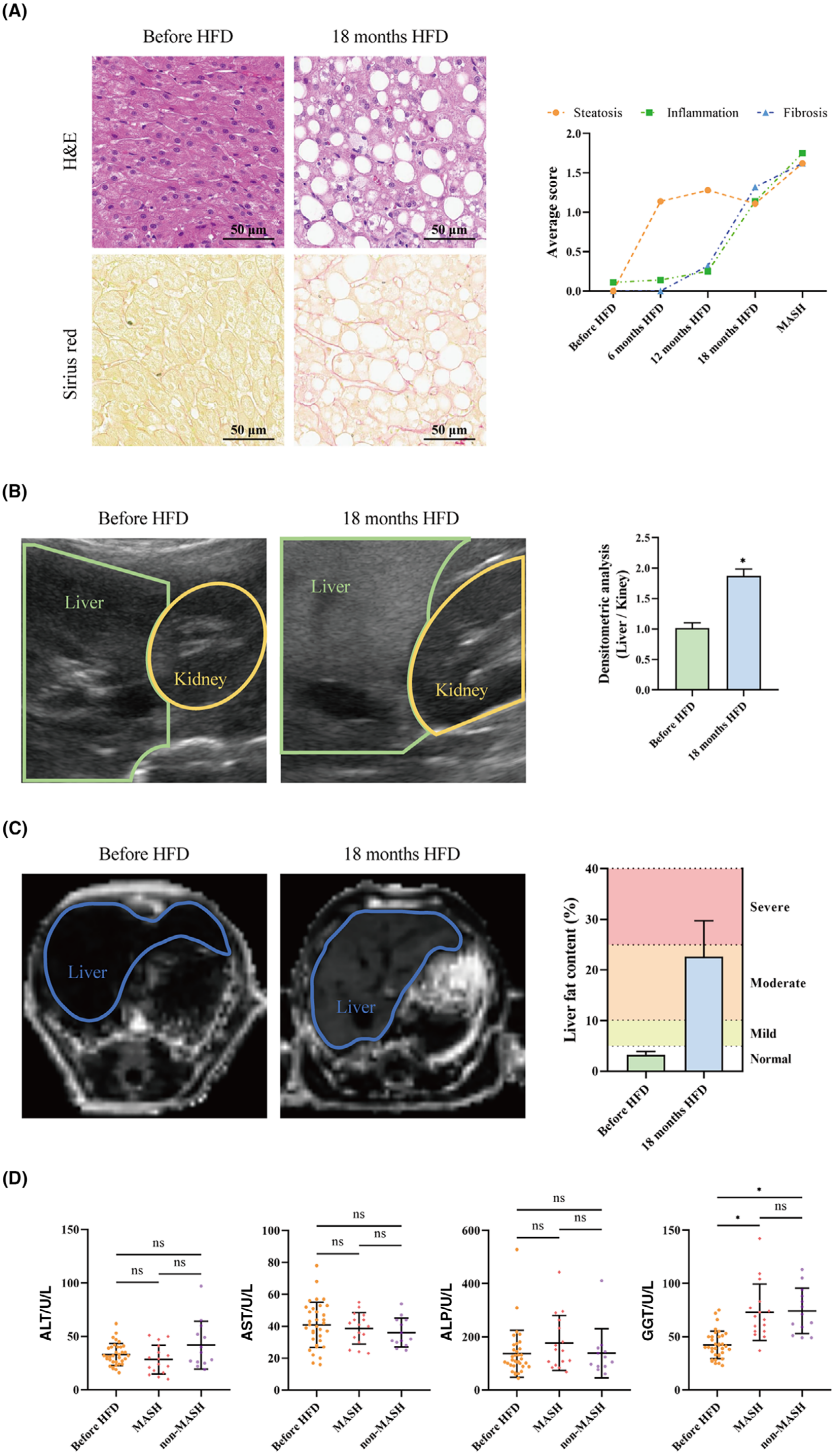
Liver tissue pathology and analysis of liver function parameters. (A) H&E staining showed hepatocytic ballooning and inflammatory infiltration. Sirus Red staining showed that the fibrotic lesions pathology score was significant increased after the HFD intervention. (B) The liver ultrasound results showed a significant increase in liver echo intensity compared to kidney as a reference. (C) The liver MRI measurement confirmed that the liver fat fraction reached 22.64% after 18 months HFD. (D) Regarding liver function parameters, GGT was significant increased after the HFD intervention, while no significant changes were observed in ALT, AST, and ALP. Data are presented as mean ± SD. Statistical significance: *p < 0.05.

Chronic consumption of a HFD has been implicated in the pathogenesis of MASH. Liver biopsy pathology confirmed that, following 18 months of continuous HFD intervention, 57.14% (16/28) of *M. fascicularis* progressed to MASH, which was histopathologically defined by the presence of steatosis, inflammatory infiltration, and hepatocytic ballooning. Furthermore, fibrotic lesions were also observed in these animals, a feature consistent with MASH. After 18 months HFD intervention, 20 *M. fascicularis* presented with inflammatory infiltration, 18 *M. fascicularis* displayed hepatocytic ballooning, and 24 *M. fascicularis* had fibrotic lesions, and all liver tissues from the *M. fascicularis* demonstrated pathological alterations. Animals meeting the diagnostic criteria were categorized into the MASH group, while all other animals were placed in the non‐MASH group. The average pathological score for the MASH group was higher than that of the other groups (Figure 2A, Table S3).

Liver ultrasound and MRI further confirmed the successful establishment of the MASH model. The liver echo intensity increased by 74.48% (Figure 2B), and the mean fat fraction increased by 19.60% after the HFD intervention (Figure 2C, Table S4), indicating a marked accumulation of liver fat. Alterations in various serum biochemical parameters indicated compromised liver function. Specifically, elevated levels of γ‐glutamyl transferase (GGT) indicate liver function damage. There was a 72.37% increase in the MASH group and a 75.43% increase in the non‐MASH group. However, no significant changes were observed in alanine transaminase (ALT), aspartate transaminase (AST), and alkaline phosphatase (ALP) levels (Figure 2D, Table S2). And after 18 months HFD intervention, the MASH group did not showed a significant difference in terms of those parameters compared to the non‐MASH group (Figure 2D, Table S2).

**FIGURE 3:**
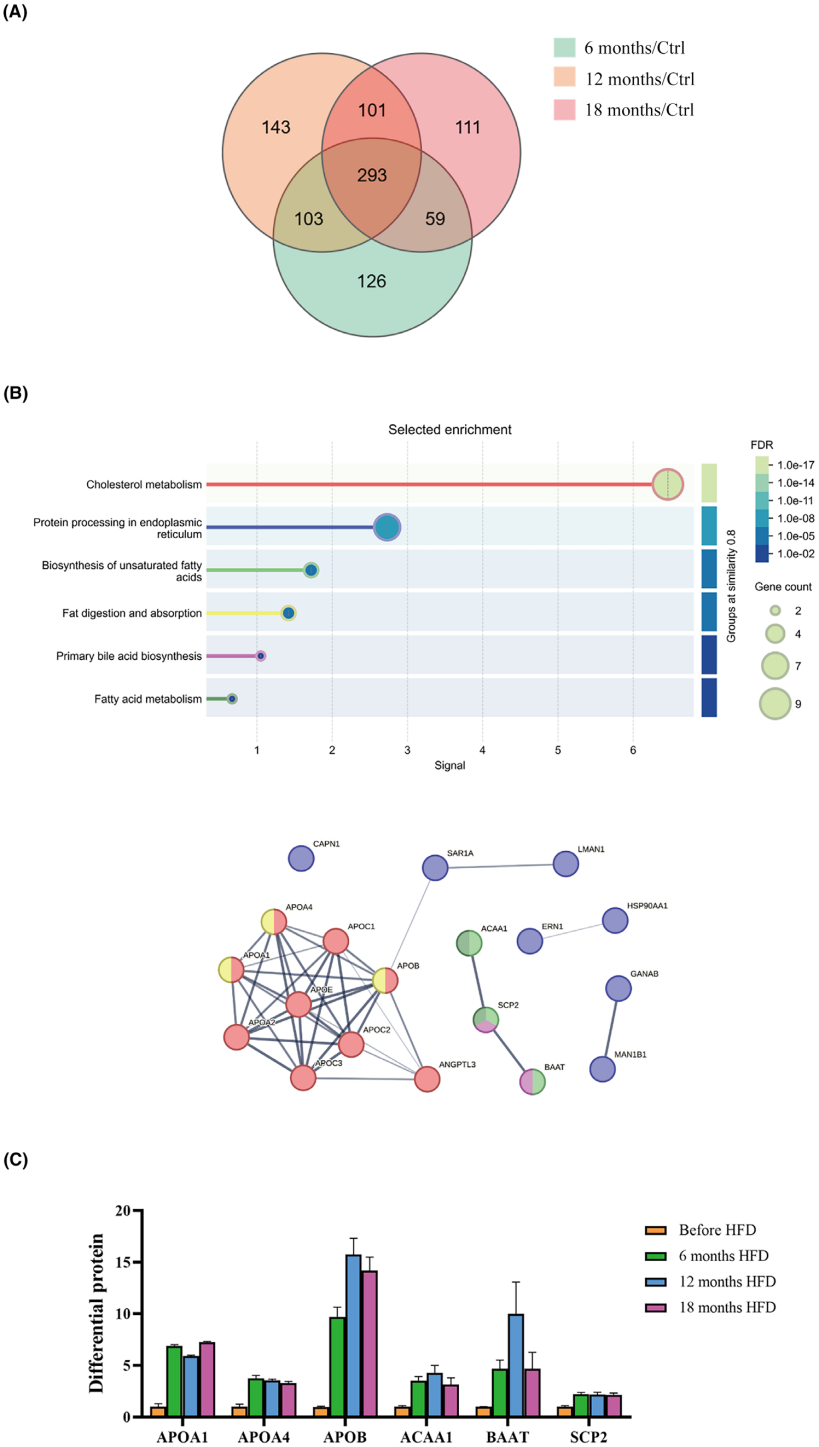
Proteomic analysis of MASH. (A) The number of MASH‐related DEPs intersected at 6, 12, and 18 months of HFD, in a Venn diagram of the three time points. (B) Analysis of common proteins after 6, 12, and 18 months intervention using the STRING database, showing which pathways were associated with MASH. (C) Statistics for key regulatory protein expression levels. Data are presented as mean ± SD. Statistical significance: *p < 0.05.

Proteomic analysis of serum samples collected at 6 months, 12 months, and 18 months HFD from *M. fascicularis* diagnosed with MASH (*n* = 16) identified 293 proteins that exhibited consistent changes across the three timepoints. Furthermore, the common proteins were subjected to analysis used the STRING database. The results showed significant enrichment in fatty acid and cholesterol metabolism pathways. Among them were 6 proteins that concurrently engaged in various signaling pathways, including Apolipoprotein A1 (APOA1), Apolipoprotein A4 (APOA4), Apolipoprotein B (APOB), Acetyl‐coA acyltransferase 1 (ACAA1), Bile acid‐CoA: amino acid N‐acyltransferase (BAAT), and Sterol carrier protein 2 (SCP2). The average fold changes in the expression levels of these proteins were as follows: APOA1 increased by 569.07%, APOA4 by 252.77%, APOB by 1221.34%, ACAA1 by 265.42%, BAAT by 544.42%, and SCP2 by 117.86% (Figure 3A–C). Mechanistically, the expression level of BAAT, a hallmark protein for MASH, showed a significant negative correlation with the degree of fibrosis, suggesting its potential involvement in the regulation of this process.

### 3.3 Prolonged HFD intervention increases the risk of potential diabetes

**FIGURE 4:**
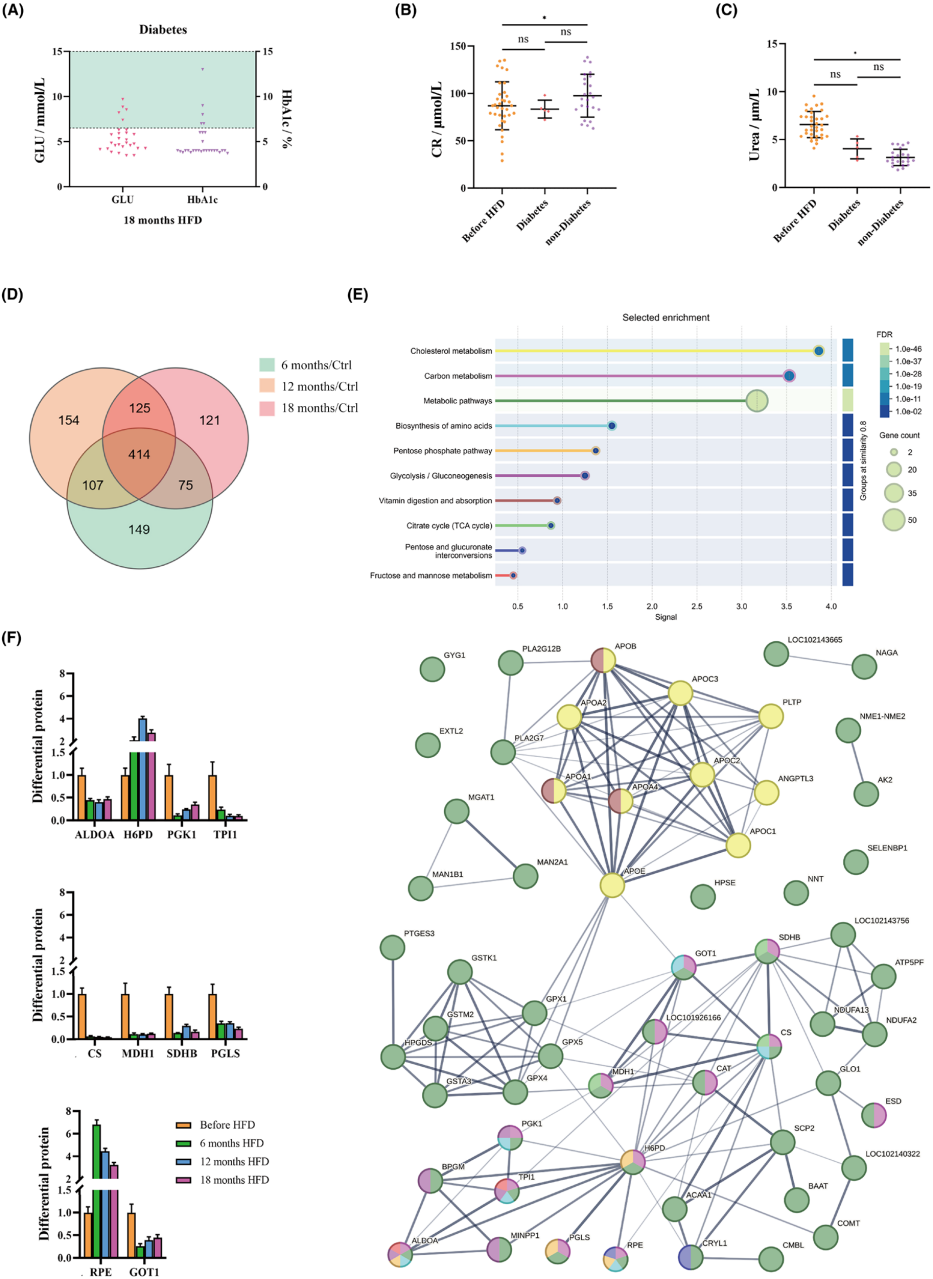
Serum biochemistry analysis and proteomic analysis of HFD‐induced diabetes. (A) Five Macaca fascicularis exhibited elevated glycemic indices: HbA1c > 6.5% and FPG > 7 mmol/L in serum biochemistry analysis. (B) Compared with before HFD levels, the CR in the non‐diabetic group significantly increased, while there was no significant difference between the diabetic group and the non‐diabetic group. (C) Compared with before HFD, the urea levels in the non‐diabetic group significantly decreased. (D) The number of diabetes‐related DEPs intersected at 6, 12, and 18 months, in a Venn diagram of the three time points. (E) Analysis of common proteins after 6, 12, and 18 months intervention using the STRING database, showing which pathways were associated with diabetes. (F) Statistics of key regulatory protein expression levels. Data are presented as Mean ± SD. Statistical significance: * (*p* < 0.05).

Chronic consumption of a HFD may contribute to the pathogenesis of diabetes. A serum biochemical analysis was conducted, and based on the assessment of FPG and HbA1c levels, which serve as two indicators for diabetes, 17.86% (5/28) of the *M. fascicularis* were diagnosed with diabetes. The results showed that FPG and HbA1c were significantly increased; FPG showed a substantial rise with a mean increase of 114.19%. Moreover, the HbA1c values of 5 *M. fascicularis* exceeded 6.5% when the FPG level was above 7 mmol/L (Figure 4A, Table S2). Those that met the criteria were classified as the diabetes group, while the remainder were assigned to the non‐diabetes group. After 18 months HFD intervention, serum creatinine (CR) levels remained stable but a marked decline in urea levels was observed. The diabetes group exhibited a 38.46% decrease, while the non‐diabetes group showed a more pronounced 52.22% decrease (Figure 4B,C). These reductions are potentially indicative of impaired urea synthesis in the liver, thereby suggesting underlying hepatic dysfunction.

Proteomic analysis of serum samples collected at 6 months, 12 months, and 18 months HFD from *M. fascicularis* diagnosed with diabetes (*n* = 5), identified 414 proteins that exhibited consistent changes across the three timepoints. The STRING database analysis results showed significant enrichment in carbon metabolism, the tricarboxylic acid cycle (TCA) cycle, and the pentose phosphate pathway. Among them were 18 proteins that concurrently engaged in various signaling pathways. Specifically, 10 of these proteins, including fructose‐1,6‐bisphosphate aldolase A (ALDOA), hexose‐6‐phosphate dehydrogenase (H6PD), phosphoglycerate kinase 1 (PGK1), triosephosphate isomerase 1 (TPI1), citrate synthase (CS), malate dehydrogenase 1 (MDH1), succinate dehydrogenase subunit B (SDHB), 6‐phosphogluconolactonase (PGLS), ribulose‐5‐phosphate‐3‐epimerase (RPE), and glutamic‐oxaloacetic transaminase 1 (GOT1), are closely linked to the glucose metabolism pathways. The observed average changes in the levels of these proteins were as follows: H6PD increased by 196.80%, RPE by 382.97%, while ALDOA decreased by 56.06%, PGK1 by 76.68%, TPI1 by 85.66%, CS by 94.79%, MDH1 by 88.72%, SDHB by 80.00%, PGLS by 68.97%, and GOT1 by 63.44% (Figure 4D–F). The downregulation of CS and MDH1 contributed to disorders in energy metabolism, while the upregulation of H6PD by 196.80% enhanced gluconeogenesis. These two factors together contributed to the disruption of blood glucose homeostasis.

### 3.4 Prolonged HFD intervention increases the risk of potential myocardial hypertrophy

**FIGURE 5:**
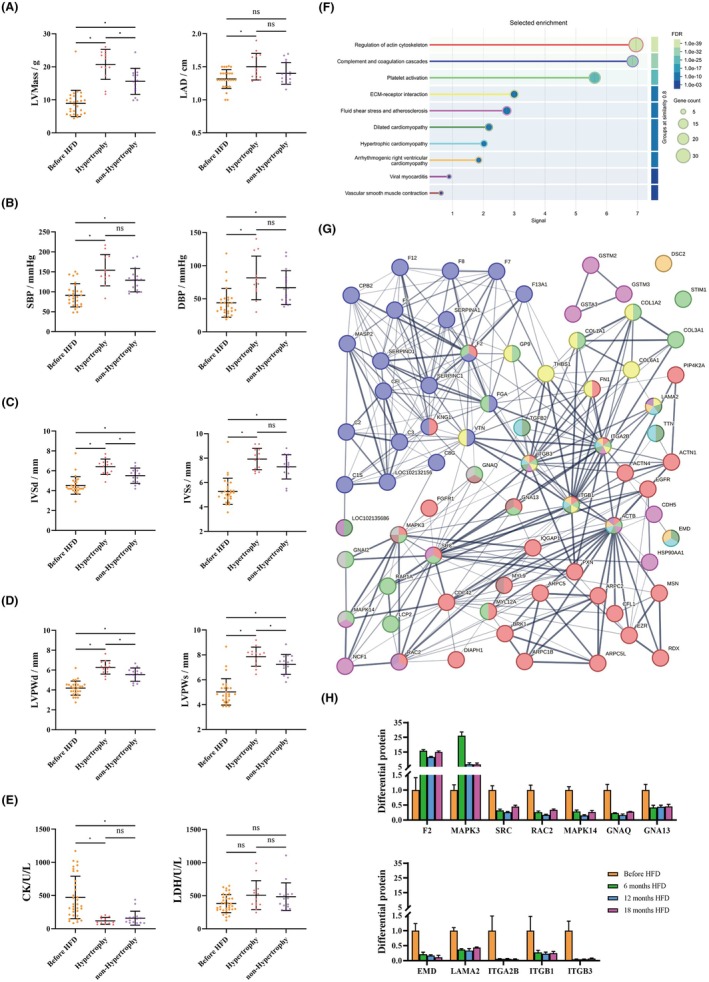
Echocardiography indices changes and proteomic analysis in HFD‐induced myocardial hypertrophy. (A–D) After the HFD intervention LVMass and LAD (A) showed significant enlargement of the heart, SBP and DBP (B) exhibited an increase, and the myocardial hypertrophy indicators IVSd, IVSs (C), LVPWd and LVPWs (D) were significant increased. (E) However, a significant reduction in CK levels was observed, whereas LDH levels remained relatively stable. (F) Analysis of proteins after 18 months intervention using the STRING database, showing which pathways were associated with myocardial hypertrophy. (G) Statistics of key regulatory protein expression levels. (H) Statistics of key regulatory protein expression levels. Data are presented as mean ± SD. Statistical significance: *p < 0.05.

Chronic consumption of a HFD may exacerbate the development of myocardial hypertrophy. Echocardiographic findings revealed signs of cardiac hypertrophy, notably a pronounced increase in cardiac size, with significant elevations in left ventricular mass (LVMass) and left atrial diameter (LAD). In the hypertrophy group, LVMass increased by 176.47% and LAD by 108.84%. By contrast, the non‐hypertrophy group exhibited far smaller increases: 14.65% for LVMass and 5.75% for LAD, respectively (Figure 5A, Table S5). Echocardiographic assessment revealed that 46.43% (13/28) *M. fascicularis* exhibited myocardial hypertrophy, as indicated by a ≥20% increase in LVMass relative to their pre‐diet baseline. Animals fulfilling the criteria were assigned to the hypertrophy group, while the remaining subjects constituted the non‐hypertrophy group. Hypertrophy led to high systolic blood pressure (SBP) and diastolic blood pressure (DBP). In the hypertrophy group, SBP increased by 74.86% and DBP by 92.06%, whereas in the non‐hypertrophy group, the increases were 46.54% for SBP and 57.43% for DBP, respectively (Figure 5B, Table S1). In addition, ventricular wall thickening was marked, with significant increases in the diastolic and systolic interventricular septum thickness (IVSd/IVSs), as well as the diastolic and systolic left ventricular posterior wall thickness (LVPW). In the hypertrophy group, IVSd increased by 43.46%, IVSs by 55.83%, LVPWd by 57.29% and LVPWs by 65.45%, whereas in the non‐hypertrophy group, the increases were 24.70% for IVSd, 44.72% for IVSs, 40.29% for LVPWd and 54.35% for LVPWs, respectively (Figure 5C,D, Table S5). Interestingly, despite the absence of myocardial injury, creatine kinase (CK) levels significantly declined following feeding, with decreases of 74.84% in the hypertrophy group and 66.27% in the non‐hypertrophy group, whereas lactate dehydrogenase (LDH) levels remained unchanged (Figure 5E). After 18 months of HFD intervention, the hypertrophy group had slightly higher echocardiography indices compared to the non‐hypertrophy group. Among them, only LVMass, IVSd, LVPWd, CK and LDH showed significant differences (Figure 5A–E).

Correlation and regression analyses were performed on two independent datasets to elucidate the intricate relationship between cardiac physiology and metabolic parameters in *M. fascicularis*. The correlation matrix indicated strong associations between BMI and various echocardiographic indices, such as LVMass, LAD, IVSd, IVSs, LVPWd, LVPWs, left ventricular internal diameter at end‐diastole (LVIDd), and stroke volume (SV). Similarly, both TG and LDL‐C showed strong correlations with EF. TCHO and LDL‐C were strongly correlated with IVSd. AST exhibited strong correlations with LVMass, IVSd, IVSs, LVPWd, and LVPWs. GGT was strongly correlated with IVSd. SBP was strongly correlated with IVSs, while DBP was strongly correlated with LVPWs. Urea was strongly correlated with LAD, and LDH with LVMass, IVSd, LVPWd, and LVPWs (Table 1). Multiple regression analysis was performed to predict echocardiographic indices based on metabolic parameters. The results showed that BMI was independently correlated with IVSd. TG was independently correlated with LVIDd. TCHO was independently correlated with IVSs. ALP was independently correlated with IVSd, left ventricular internal diameter at end‐systole (LVIDs), and ejection fraction (EF). GGT was independently correlated with IVSd. Both SBP and DBP were independently correlated with LVPWs. Urea was independently correlated with LVMass, IVSs, and LVPWs. LDH was independently correlated with IVSd, LVIDs, and EF (Table 2). Table 3 results indicated that changes in BMI were positively correlated with changes in LVMass, IVSs, LVIDd, and SV. Additionally, AST was positively correlated with changes in IVSd, LVPWd, and LVPWs.

**TABLE 1:** Correlation coefficients (*r*) between echocardiographic indices and metabolic parameters.

	LVMass (g)	LAD (mm)	IVSd (mm)	IVSs (mm)	LVPWd (mm)	LVPWs (mm)	LVIDd (mm)	LVIDs (mm)	EF (%)	SV (mL)
BMI (kg/m^2^)	0.547*	0.345*	0.732*	0.562*	0.551*	0.681*	0.357*	0.045	0.227	0.480*
FPG (mmol/L)	−0.047	0.047	−0.037	0.040	−0.010	−0.033	−0.022	−0.013	−0.006	−0.049
TG (mmol/L)	0.010	0.233	0.208	0.129	0.265	0.230	0.079	−0.215	0.356*	0.258
TCHO (mmol/L)	−0.164	0.021	−0.354*	−0.186	−0.091	−0.232	0.057	0.205	−0.270	−0.063
HDL‐C (mmol/L)	−0.147	0.078	−0.199	−0.133	−0.016	−0.135	−0.024	0.079	−0.167	−0.094
LDL‐C (mmol/L)	−0.063	0.094	−0.348*	−0.222	−0.186	−0.208	0.146	0.328	−0.362*	−0.026
ALT (U/L)	−0.108	−0.157	−0.153	−0.066	−0.071	−0.083	−0.026	−0.058	0.060	0.010
AST (U/L)	−0.343*	−0.265	−0.452*	−0.392*	−0.383*	−0.411*	−0.174	0.003	−0.180	−0.232
ALP (U/L)	−0.055	−0.028	−0.202	−0.052	−0.081	−0.176	0.002	0.114	−0.192	−0.093
GGT (U/L)	−0.254	0.086	−0.377*	−0.237	−0.120	−0.187	−0.118	−0.096	0.030	−0.086
SBP (mmHg)	0.208	−0.125	0.233	0.349*	0.182	0.300	0.162	−0.013	0.161	0.233
DBP (mmHg)	0.187	−0.016	0.208	0.218	0.213	0.369*	0.095	−0.039	0.144	0.186
CR (μmol/L)	−0.133	−0.107	−0.145	−0.045	0.004	−0.104	−0.053	−0.029	−0.035	−0.040
UREA (μmol/L)	−0.168	−0.346*	−0.171	−0.130	−0.178	−0.099	−0.166	−0.026	−0.120	−0.174
CK (U/L)	−0.294	−0.314	−0.168	−0.267	−0.314	−0.308	−0.196	−0.135	−0.012	−0.155
LDH (U/L)	−0.338*	−0.318	−0.466*	−0.318	−0.364*	−0.460*	−0.038	0.020	−0.089	−0.075

* *p* < 0.05.

**TABLE 2:** Coefficients of multiple linear regression analysis of echocardiographic indices with metabolic parameters.

	LVMass (g)	LAD (mm)	IVSd (mm)	IVSs (mm)	LVPWd (mm)	LVPWs (mm)	LVIDd (mm)	LVIDs (mm)	EF (%)	SV (mL)
BMI (kg/m^2^)	0.032	−0.516	2.810*	−1.395	−0.009	1.297	1.721	−1.637	−1.635	−0.691
FPG (mmol/L)	−1.105	1.145	−0.489	1.780	−1.176	0.819	0.710	−0.463	−0.661	−0.762
TG (mmol/L)	0.630	−0.093	−0.519	−0.991	1.645	−0.536	1.902	−2.116*	−1.867	−0.509
TCHO (mmol/L)	−1.810	1.262	−1.606	2.451*	−0.720	1.084	0.537	−0.487	−1.101	−0.006
HDL‐C (mmol/L)	−1.577	1.329	−0.669	1.906	−0.572	0.980	0.889	−0.915	−1.471	−0.229
LDL‐C (mmol/L)	−0.705	1.638	−1.414	1.427	−1.610	0.781	0.661	−0.557	−0.924	−0.395
ALT (U/L)	0.543	−0.749	−1.279	−0.306	0.300	−0.279	0.326	−0.366	−0.168	−0.049
AST (U/L)	0.354	0.050	−1.338	0.214	−0.576	−0.380	0.566	−0.962	−1.164	0.309
ALP (U/L)	0.535	1.598	−2.249*	1.457	−0.841	−0.823	1.997	−2.266*	−2.389*	−0.894
GGT (U/L)	0.648	1.302	−2.709*	0.491	0.467	−0.800	0.471	−0.983	−1.004	0.410
SBP (mmHg)	−1.796	−1.259	−0.142	1.788	−1.143	2.222*	0.428	0.035	−0.183	−0.457
DBP (mmHg)	−1.401	−1.508	0.604	0.200	−0.312	2.312*	−0.718	0.955	0.732	0.476
CR (μmol/L)	−0.637	0.150	−1.182	1.215	0.052	0.194	0.723	−1.038	−1.408	0.315
UREA (μmol/L)	−3.135*	−1.275	0.915	2.090*	−1.909	3.254*	−1.690	1.675	0.669	1.568
CK (U/L)	0.307	−0.601	0.362	−0.347	−0.452	−0.499	0.536	−1.152	−1.469	0.780
LDH (U/L)	0.658	−0.551	−2.572*	0.787	0.631	−1.540	1.794	−2.249*	−2.315*	−0.067

* *p* < 0.05.

**TABLE 3:** Correlation coefficients (*r*) between metabolic indexes changes and cardiac indexes changes.

	ΔLVMass (g)	ΔLAD (mm)	ΔIVSd (mm)	ΔIVSs (mm)	ΔLVPWd (mm)	ΔLVPWs (mm)	ΔLVIDd (mm)	ΔLVIDs (mm)	ΔEF (%)	ΔSV (mL)
ΔBMI (kg/m^2^)	0.404*	0.283	0.252	0.393*	0.322	0.231	0.375*	0.095	0.245	0.373*
ΔFPG (mmol/L)	−0.018	−0.173	0.041	0.049	−0.010	0.162	−0.239	−0.005	−0.118	−0.306
ΔTG (mmol/L)	−0.118	−0.176	0.061	0.178	0.134	0.000	−0.366	−0.008	−0.302	−0.415*
ΔTCHO (mmol/L)	0.032	0.258	−0.004	0.132	0.062	0.029	0.092	0.357	−0.332	−0.034
ΔHDL‐C (mmol/L)	0.118	0.270	0.216	0.227	0.203	0.133	0.003	0.234	−0.279	−0.070
ΔLDL‐C (mmol/L)	−0.005	0.230	−0.087	0.096	0.041	−0.023	0.064	0.336	−0.328	−0.052
ΔALT (U/L)	−0.321	0.217	−0.348	−0.343	−0.240	−0.355	−0.096	−0.054	0.061	−0.050
ΔAST (U/L)	−0.318	0.061	−0.531*	−0.275	−0.435*	−0.478*	0.039	0.127	−0.071	0.024
ΔALP (U/L)	−0.283	−0.036	−0.261	−0.339	−0.301	−0.331	−0.223	−0.020	−0.278	−0.202
ΔGGT (U/L)	−0.261	−0.051	−0.148	−0.055	−0.197	−0.171	−0.279	0.058	−0.257	−0.380*
ΔSBP (mmHg)	0.214	0.040	0.068	0.043	−0.030	0.081	0.305	0.239	−0.033	0.286
ΔDBP (mmHg)	0.127	−0.035	0.042	−0.051	−0.120	0.043	0.184	0.117	0.023	0.202
ΔCR (μmol/L)	−0.113	−0.015	−0.014	−0.049	−0.055	0.040	0.063	−0.426*	0.483*	0.321
ΔUREA (μmol/L)	−0.163	−0.224	−0.088	−0.078	−0.124	0.080	−0.312	−0.060	−0.130	−0.344
ΔCK (U/L)	−0.282	−0.217	−0.160	−0.115	−0.279	−0.129	−0.178	−0.123	−0.019	−0.143
ΔLDH (U/L)	−0.068	0.201	−0.328	−0.135	−0.160	−0.360	0.100	0.141	−0.042	0.023

* *p* < 0.05.

Proteomic analysis of serum samples collected from *M. fascicularis* diagnosed with myocardial hypertrophy (*n* = 13) after 18 months of HFD identified 830 differentially expressed proteins. The STRING database analysis results showed significant enrichment in pathways related to platelet activation, regulation of the actin cytoskeleton, and lipid metabolism and atherosclerosis. Among them were 26 proteins that concurrently engaged in various signaling pathways. Specifically, 12 of these proteins, including Coagulation factor II (F2), Mitogen‐activated protein kinase 3 (MAPK3), SRC proto‐oncogene, non‐receptor tyrosine kinase (SRC), Rac family small GTPase 2 (RAC2), mitogen‐activated protein kinase 14 (MAPK14), G protein subunit alpha Q (GNAQ), G protein subunit alpha 13 (GNA13), emerin (EMD), Laminin subunit alpha 2 (LAMA2), Integrin subunit alpha 2b (ITGA2B), integrin subunit beta 1 (ITGB1) and Integrin subunit beta 3 (ITGB3), are closely linked to atherosclerosis and myocardial injury. The average changes in protein expression levels were as follows: F2 showed a 1316.85% increase, MAPK3 a 1210.69% increase; while SRC decreased by 65.58%, RAC2 by 74.51%, MAPK14 by 76.33%, GNAQ by 77.37%, GNA13 by 56.29%, EMD by 83.41%, LAMA2 by 62.12%, ITGA2B by 94.72%, ITGB1 by 74.97%, and ITGB3 by 94.79% (Figure 5F–H). The identified proteins showed that the downregulation of several proteins, including SRC, MAPK14, EMD, and ITGB1, was associated with the hypertrophic phenotype, suggesting they may play a protective role.

## 4 DISCUSSION

Numerous studies have demonstrated that long‐term high‐fat diet (HFD) can exert comprehensive adverse effects on the body.^16^, ^17^ Due to the complexity of the pathogenesis of HFD‐induced metabolic‐related diseases,^18^, ^19^, ^20^ and limited human‐relevant animal models, only one MASH drug is globally approved.^21^ NHPs, as humans' closest relatives, can best recapitulate human metabolic disease progression and aid in drug development and disease prevention.^22^ Current NHP studies focus predominantly on fatty liver,^23^, ^24^, ^25^, ^26^ with few assessing multi‐organ risks. This study demonstrates that 18‐month HFD progressively induces MASH, myocardial hypertrophy, and diabetes in *M. fascicularis*, providing critical insights into multisystem metabolic disease mechanisms.

Clinical studies indicate that compensatory effects limit MAFLD to MASH progression to 20%–30% in humans.^27^ Similarly, 57.14% of *M. fascicularis* developed MASH after 18 months of HFD—lower than expected. Human studies show even a 5‐day HFD rapidly increases liver fat,^28^ while MAFLD‐to‐MASH progression typically takes 3.5–5 years.^27^ In this study, HFD‐fed *M. fascicularis* showed mild steatosis and inflammation at 12 months, with significant steatosis and fibrosis at 18 months. It is generally recognized that 1 year of *M. fascicularis* life is equivalent to 3 years in humans.^29^ Thus, the 18‐month experimental period in *M. fascicularis* model translates to roughly 4–5 years of human time, which is consistent with the progression rate of human MASH. This study also found that lipid metabolism markers decreased at 18 months compared to 12 months, possibly due to reduced HFD intake from waning interest in later stages. Proteomic analysis identified potential MASH biomarkers, revealing several key associations: Elevated APOA1 levels were associated with reduced NAFLD risk,^30^ while APOA4 and APOB showed no clinical association with MAFLD. However, an increased ApoB/A1 ratio was found to elevate MAFLD risk, aligning with the findings of this study.^31^ Furthermore, ACAA1 was confirmed as a key regulator in NAFLD,^32^ BAAT upregulation demonstrated links to obesity,^33^ and SCP2 was implicated in MAFLD pathogenesis through modulation of lipid metabolism.^34^ Critically, long‐term HFD exposure in *M. fascicularis* recapitulated human MASH phenotypes, reinforcing the translational relevance of these biomarkers.

Hyperglycemia and hyperlipidemia frequently co‐occur due to metabolic interconversion through the TCA cycle, while MASH and diabetes demonstrate bidirectional exacerbation.^35^, ^36^, ^37^ This study confirms that HFD induces hyperglycemia and hyperlipidemia in *M. fascicularis*, consistent with metabolic syndrome.^38^ Proteomic analysis identified three key glucose‐disorder biomarkers: CS and MDH1 were downregulated. These associations are linked to mitochondrial damage,^39^ reduced exercise capacity,^40^ and diabetic hyperglycemia.^41^, ^42^ In contrast, H6PD was upregulated, correlating with T2D.^43^ Concurrently, decreased PGK1^44^ and TPI1^45^ in diabetes, alongside GOT1 reduction in diabetic models,^46^ were observed. However, SDHB, PGLS, and RPE showed no association with diabetes. Collectively, suppressed CS and MDH1 likely impairs energy metabolism, while elevated H6PD may promote gluconeogenesis, synergistically disrupting glucose homeostasis.

Clinical studies confirm MASH is linked to cardiac structural remodeling and dysfunction.^47^, ^48^ Mouse models demonstrate long‐term HFD induces myocardial hypertrophy,^49^ while *M. fascicularis* studies show an 8‐month HFD reduces cardiac contractility.^50^ In this 18‐month HFD study, 46.43% of *M. fascicularis* developed myocardial hypertrophy, establishing long‐term HFD as a significant risk factor for cardiac pathology.

Proteomic analysis identified SRC, MAPK14, EMD, and ITGB1 as potential mediators of HFD‐induced myocardial hypertrophy. A literature review established that MAPK3 serves as a therapeutic target for hypertrophy,^51^, ^52^ while SRC drives cardiac hypertrophy through integrin and FAK signaling regulation.^53^ ITGB1 suppresses hypertrophy by inhibiting cardiomyocyte autophagy^54^ and dysregulating AKT signaling.^55^ In contrast, GNAQ promotes human myocardial hypertrophy,^56^ whereas GNA13 may confer cardioprotection via RHOA inhibition.^57^ EMD maintains nuclear integrity and calcium dynamics in cardiomyocytes.^58^ Although F2 shows no coronary disease association, it interacts with coagulation factors IX and XI,^59^ and converts to thrombin.^60^ Limited evidence links RAC2 and MAPK14 to hypertrophy, while LAMA2 mutations associate with cardiomyopathy^61^; ITGA2B and ITGB3 mutations conversely affect platelet function in thrombotic disorders.^62^ Critically, this MASH model successfully recapitulated myocardial hypertrophy, enabling multisystemic metabolic syndrome research.

In summary, metabolic dysfunction is characterized by dysregulation in energy and fatty acid metabolism, including complex processes such as glycogenesis, glycogenolysis, and gluconeogenesis. Disruptions in fatty acid metabolism can lead to oxidative stress and lipid accumulation, resulting in pathophysiological states such as obesity, dyslipidemia, reduced glucose tolerance, and insulin resistance. These metabolic derangements can manifest as MASH, cardiac hypertrophy, and diabetes mellitus in humans. This study used a long‐term HFD‐fed *M. fascicularis* model of metabolic syndrome to effectively simulate human clinical characteristics and reproduce three metabolic syndrome‐related diseases: MASH, cardiac hypertrophy, and diabetes. It reveals that HFD can induce multi‐organ dysfunction and its potential mechanisms, providing a reference for the treatment and prevention of related diseases.

## AUTHOR CONTRIBUTIONS

**Hongyi Chen:** Data curation; formal analysis; software; validation; writing – original draft. **Wei Liu:** Resources. **Dan Zhou:** Data curation; formal analysis. **Shuhua Liu:** Investigation; methodology. **Yalun Guan:** Investigation; methodology. **Zongyu Miao:** Investigation; methodology. **Lei Cai:** Funding acquisition; writing – review and editing. **Xuejiao Li:** Investigation; methodology. **Yunfeng Li:** Investigation; methodology. **Zhongqiang Huang:** Investigation; methodology. **Yi Jin:** Funding acquisition; resources. **Ge Li:** Conceptualization; funding acquisition; project administration; supervision. **Yu Zhang:** Conceptualization; funding acquisition; project administration; supervision; writing – review and editing.

## FUNDING INFORMATION

This work was supported by National Key Research and Development Program of China (2021YFF0702200), Guangdong S&T programme (2009A081000002, 2023B0303040004), Science and Technology Projects in Guangzhou (202206010084, 202206010197, 202206060002), Technology Planning Project of Linzhi (2023‐YZ‐01).

## CONFLICT OF INTEREST STATEMENT

The authors declare no conflict of interest.

## ETHICS STATEMENT

Animals were used after approval from the Institutional Animal Care and Use Committee of Guangdong Provincial Biotechnology Research Institute (IACUC no. 2021147, AAALAC accredited).

## Supporting information


**TABLE S1.** Statistical data of BMI and BP.
**TABLE S2**. Statistical data of blood biochemical analysis.
**TABLE S3**. Liver pathology score.
**TABLE S4**. Liver segmentation and fat fraction.
**TABLE S5**. Statistical data of echocardiographic parameters.

## Data Availability

The mass spectrometry proteomics data have been deposited to the iProX with the dataset identifer PXD058881.
