# Supplementary material for: Chronic high‐fat diet induces multi‐organ dysfunction and metabolic homeostasis disruption in Macaca fascicularis
Source: Animal Model Exp Med. 2026 Jan 14;9(1):193–206. doi: 10.1002/ame2.70124 (PMC12907977; doi:10.1002/ame2.70124)
Supplement: Supplementary file 1 — TABLE S1. Statistical data of BMI and BP. TABLE S2. Statistical data of blood biochemical analysis. TABLE S3. Liver pathology score. TABLE S4. Liver segmentation and fat fraction. TABLE S5. Statistical data of echocardiographic parameters. [file AME2-9-193-s001.docx]

**Supplementary material**

Table.S1 Statistical data of BMI and BP

|  | Before HFD (n=35) | 6 months HFD (n=35) | 12 months HFD (n=34) | 18 months HFD (n=28) |
| --- | --- | --- | --- | --- |
| Weight (kg) | 7.48 ± 1.88 | 9.16 ± 2.65^*^ | 9.86 ± 2.93^*^ | 9.60 ± 3.08^*^ |
| BMI | 36.70 ± 8.38 | 44.64 ± 11.95^*^ | 48.53 ± 13.01^*^ | 46.97 ± 13.75^*^ |
| SBP (mmHg) | 88.38 ± 27.54 | 129.41 ± 26.78^*^ | 124.13 ± 32.56^*^ | 138.96 ± 35.93^*^ |
| DBP (mmHg) | 42.45 ± 20.41 | 69.88 ± 18.39^*^ | 62.58 ± 18.19^*^ | 72.79 ± 30.46^*^ |

Note: **P* < 0.05, compared with Before HFD. Data are presented as Mean ± SD.

Table.S2 Statistical data of blood biochemical analysis

|  | Before HFD (n=35) | 6 months HFD (n=35) | 12 months HFD (n=34) | 18 months HFD (n=28) |
| --- | --- | --- | --- | --- |
| ALT (U/L) | 32.80 ± 10.23 | 39.44 ± 18.25^*^ | 39.79 ± 33.06 | 34.37 ± 18.94 |
| AST (U/L) | 41.77 ± 15.77 | 39.61 ± 14.05 | 42.05 ± 19.34 | 37.51 ± 9.42 |
| AST/ALT | 1.34 ± 0.60 | 1.24 ± 0.85 | 1.24 ± 0.52 | 1.35 ± 0.64 |
| ALP (U/L) | 168.63 ± 201.08 | 167.80 ± 59.58^*^ | 216.44 ± 150.16^*^ | 190.96 ± 190.72 |
| GGT (U/L) | 43.94 ± 16.03 | 74.58 ± 35.59^*^ | 73.44 ± 31.71^*^ | 73.51 ± 23.82^*^ |
| TP (g/L) | 56.12 ± 10.32 | 78.94 ± 10.31^*^ | 88.33 ± 20.57^*^ | 81.59 ± 9.42^*^ |
| ALB (g/L) | 30.53 ± 5.68 | 39.58 ± 4.89^*^ | 42.35 ± 9.66^*^ | 39.31 ± 5.42^*^ |
| TBIL (μmol/L) | 3.03 ± 1.67 | 2.57 ± 1.39 | 2.41 ± 0.98^*^ | 3.27 ± 0.91 |
| DBIL (μmol/L) | 1.11 ± 0.42 | 0.64 ± 0.36^*^ | 0.80 ± 0.61^*^ | 2.06 ± 0.52^*^ |
| UREA (μmol/L) | 6.64 ± 1.41 | 4.08 ± 1.68^*^ | 4.67 ± 1.16^*^ | 4.01 ± 3.95^*^ |
| CR (μmol/L) | 85.83 ± 25.85 | 91.11 ± 19.32 | 94.47 ± 30.92 | 95.10 ± 21.50 |
| FPG (mmol/L) | 3.10 ± 1.18 | 5.53 ± 3.53^*^ | 6.98 ± 3.29^*^ | 6.64 ± 6.50^*^ |
| TG (mmol/L) | 0.43 ± 0.17 | 1.28 ± 1.13^*^ | 1.56 ± 1.98^*^ | 1.86 ± 1.77^*^ |
| TCHO (mmol/L) | 1.86 ± 0.41 | 11.89 ± 5.45^*^ | 14.64 ± 7.96^*^ | 10.25 ± 6.85^*^ |
| HDL-C (mmol/L) | 0.67 ± 0.18 | 1.71 ± 0.49^*^ | 3.01 ± 1.53^*^ | 2.05 ± 0.98^*^ |
| LDL-C (mmol/L) | 0.79 ± 0.28 | 6.65 ± 3.18^*^ | 7.91 ± 4.65^*^ | 5.39 ± 4.05^*^ |
| CK (U/L) | 541.19 ± 450.21 | 111.61 ± 32.49^*^ | 156.11 ± 86.98^*^ | 141.20 ± 85.93^*^ |
| LDH (U/L) | 385.22 ± 135.70 | 393.50 ± 107.42 | 497.20 ± 194.24^*^ | 495.96 ± 209.72^*^ |
| NT-proBNP (pg/mL) | <10 | <10 | - | - |
| Ca (mmol/L) | 2.03 ± 0.28 | 2.54 ± 0.21^*^ | 3.04 ± 0.51^*^ | 2.52 ± 0.13^*^ |
| P (mmol/L) | 1.30 ± 0.53 | 1.79 ± 0.54^*^ | 2.05 ± 0.52^*^ | 1.38 ± 0.49 |
| Na (mmol/L) | 135.68 ± 10.50 | 145.18 ± 7.37^*^ | 162.56 ± 28.50^*^ | 150.04 ± 5.60^*^ |
| K (mmol/L) | 4.31 ± 0.97 | 4.84 ± 0.67^*^ | 5.50 ± 0.93^*^ | 5.22 ± 0.66^*^ |
| Cl (mmol/L) | 101.09 ± 5.57 | 100.89 ± 3.63 | 106.81 ± 11.20^*^ | 109.75 ± 3.77^*^ |
| HbA1c (%) | - | - | - | 5.03 ± 2.09 |

Note: **P* < 0.05, compared with Before HFD. Data are presented as Mean ± SD.

Table.S3 Liver pathology score

|  | Steatosis | | | | Inflammation | | | | Fibrosis | | | | |
| --- | --- | --- | --- | --- | --- | --- | --- | --- | --- | --- | --- | --- | --- |
| Score | 0 | 1 | 2 | 3 | 0 | 1 | 2 | 3 | 0 | 1 | 2 | 3 | 4 |
| Before HFD (n=28) | 28 | 0 | 0 | 0 | 26 | 1 | 1 | 0 | 28 | 0 | 0 | 0 | 0 |
| Average score | 0 | | | | 0.11 | | | | 0 | | | | |
| 6 months HFD (n=35) | 13 | 12 | 7 | 2 | 31 | 2 | 1 | 0 | 34 | 0 | 0 | 0 | 0 |
| Average score | 1.14 | | | | 0.14 | | | | 0 | | | | |
| 12 months HFD (n=34) | 8 | 18 | 6 | 2 | 28 | 5 | 1 | 0 | 27 | 5 | 2 | 0 | 0 |
| Average score | 1.28 | | | | 0.25 | | | | 0.32 | | | | |
| 18 months HFD (n=28) | 10 | 8 | 7 | 3 | 8 | 12 | 4 | 4 | 4 | 11 | 13 | 0 | 0 |
| Average score | 1.11 | | | | 1.14 | | | | 1.32 | | | | |
| MASH (n=16) | 3 | 5 | 6 | 2 | 3 | 5 | 4 | 4 | 1 | 5 | 10 | 0 | 0 |
| Average score | 1.62 | | | | 1.75 | | | | 1.62 | | | | |

Note: Steatosis was scored from 0-3 (0: <5% steatosis; 1: 5-33%; 2: 34-66%; 3: >67%). Lobular inflammation was scored from 0-3 based on foci of inflammation counted at 20X (0: none, 1: <2 foci; 2: 2-4 foci; 3: >4 foci). Hepatic fibrosis was scored from 0-4 (0: no fibrosis; 1: perisinusoidal or portal fibrosis; 2: perisinusoidal and portal fibrosis; 3: bridging fibrosis; 4: cirrhosis).

Table.S4 Liver segmentation and fat fraction

|  | Before HFD (n=3) | 18 months HFD (n=6) |
| --- | --- | --- |
| Ⅰ（%） | 3.52 ± 1.44 | 23.10 ± 7.65 |
| Ⅱ（%） | 3.81 ± 1.02 | 21.46 ± 7.06 |
| Ⅲ（%） | 5.75 ± 2.78 | 20.98 ± 7.73 |
| Ⅳ（%） | 3.35 ± 0.89 | 21.46 ± 6.64 |
| Ⅴ（%） | 2.50 ± 0.54 | 23.27 ± 6.24 |
| Ⅵ（%） | 2.27 ± 1.02 | 24.14 ± 7.47 |
| Ⅶ（%） | 2.17 ± 0.65 | 23.66 ± 7.61 |
| Ⅷ（%） | 2.56 ± 0.26 | 23.04 ± 7.04 |
| 全肝（%） | 3.24 ± 0.63 | 22.64 ± 7.06 |

Note: The grading of steatosis based on fat fraction is as follows: Normal: <5%, Mild: 5-10%, Moderate: 10-25%, Severe: >25%. Data are presented as Mean ± SD.

Table.S5 Statistical data of echocardiographic parameters

|  | Before HFD (n=35) | 6 months HFD (n=35) | 12 months HFD (n=34) | 18 months HFD (n=28) |
| --- | --- | --- | --- | --- |
| IVSd (mm) | 4.45 ± 0.82 | 5.89 ± 1.01^*^ | 6.15 ± 0.74^*^ | 5.93 ± 0.88^*^ |
| IVSs (mm) | 5.33 ± 1.00 | 8.06 ± 1.17^*^ | 7.84 ± 0.82^*^ | 7.64 ± 0.97^*^ |
| LVIDd (mm) | 19.97 ± 2.09 | 18.72 ± 3.05^*^ | 17.31 ± 2.41^*^ | 17.83 ± 2.63^*^ |
| LVIDs (mm) | 12.11 ± 1.94 | 11.45 ± 2.58^*^ | 10.83 ± 1.80^*^ | 11.32 ± 1.76^*^ |
| LVPWd (mm) | 4.22 ± 0.67 | 5.89 ± 0.91^*^ | 5.93 ± 0.88^*^ | 5.89 ± 0.75^*^ |
| LVPWs (mm) | 5.01 ± 0.95 | 7.77 ± 0.88^*^ | 7.64 ± 0.76^*^ | 7.59 ± 0.85^*^ |
| EDV (mL) | 12.94 ± 3.29 | 11.27 ± 4.58^*^ | 9.10 ± 3.06^*^ | 9.87 ± 3.61^*^ |
| ESV (mL) | 3.60 ± 1.55 | 3.28 ± 2.13^*^ | 2.70 ± 1.14^*^ | 3.02 ± 1.28 |
| EF (%) | 72.42 ± 7.44 | 71.40 ± 10.26 | 70.49 ± 6.93 | 68.26 ± 10.60^*^ |
| SV (mL) | 9.31 ± 2.28 | 7.99 ± 3.19^*^ | 6.40 ± 2.26^*^ | 6.85 ± 2.97^*^ |
| FS (%) | 39.48 ± 6.23 | 38.88 ± 8.54 | 37.42 ± 5.50 | 36.11 ± 7.74^*^ |
| LVMass (g) | 8.87 ± 3.58 | 23.83 ± 6.00^*^ | 17.74 ± 4.54^*^ | 17.96 ± 4.90^*^ |
| LAD (cm) | 1.30 ± 0.13 | 1.42 ± 0.17^*^ | 1.44 ± 0.20^*^ | 1.43 ± 0.19^*^ |
| E wave (m/s) | 0.68 ± 0.13 | 0.62 ± 0.14 | 0.60 ± 0.19^*^ | 0.68 ± 0.15 |
| A wave (m/s) | 0.61 ± 0.20 | 0.62 ± 0.20 | 0.50 ± 0.11^*^ | 0.66 ± 0.17 |
| E/A ratio | 1.18 ± 0.39 | 1.08 ± 0.38 | 1.22 ± 0.40 | 1.07 ± 0.25 |
| S’ medial (m/s) | 0.06 ± 0.01 | 0.04 ± 0.01^*^ | 0.05 ± 0.00^*^ | 0.07 ± 0.02 |
| E’ medial (m/s) | 0.07 ± 0.02 | 0.05 ± 0.02^*^ | 0.05 ± 0.02^*^ | 0.06 ± 0.02 |
| E/E’ medial | 9.71 ± 3.60 | 12.03 ± 4.39 | 11.37 ± 3.72 | 10.73 ± 4.22 |
| E’ lateral (m/s) | 0.10 ± 0.02 | 0.08 ± 0.02^*^ | 0.07 ± 0.02^*^ | 0.08 ± 0.02^*^ |
| E/E’ lateral | 7.02 ± 2.08 | 7.63 ± 3.08 | 7.69 ± 1.84^*^ | 7.10 ± 2.04 |
| LAV-A4C (mL) | 3.04 ± 0.75 | 3.48 ± 1.00^*^ | 3.42 ± 1.40 | 3.45 ± 1.30 |

Note: **P* < 0.05, compared with Before HFD. Data are presented as Mean ± SD.
